# Modulating N1 and N2 neutrophils in breast cancer: potential therapeutic approaches – a narrative review

**DOI:** 10.1097/MS9.0000000000004531

**Published:** 2025-12-16

**Authors:** Emmanuel Ifeanyi Obeagu

**Affiliations:** a Department of Biomedical and Laboratory Science, Africa University, Mutare, Zimbabwe; b The Department of Molecular Medicine and Haematology, School of Pathology, Faculty of Health Sciences, University of the Witwatersrand, Johannesburg, South Africa

**Keywords:** breast cancer, N1 neutrophils, N2 neutrophils, therapeutic modulation, tumor microenvironment

## Abstract

Neutrophils play a dual role in breast cancer progression, acting as anti-tumorigenic (N1) or pro-tumorigenic (N2) subtypes based on cues from the tumor microenvironment (TME). This review examines the mechanisms driving neutrophil polarization, focusing on how the TME promotes N2 dominance to support tumor growth, angiogenesis, and metastasis. Therapeutic strategies targeting these processes, including cytokine modulation, immune checkpoint inhibitors, and small-molecule drugs, are discussed. Emerging insights into neutrophil biology highlight the potential to reprogram N2 neutrophils into N1 phenotypes, offering promising avenues for enhancing cancer immunotherapy and improving breast cancer outcomes. Neutrophils are critical components of the tumor microenvironment, influencing breast cancer progression through distinct subtypes: anti-tumorigenic N1 and pro-tumorigenic N2 neutrophils. This review delves into the polarization of neutrophils by tumor-derived factors and their contrasting roles in breast cancer biology. N2 neutrophils drive angiogenesis, immunosuppression, and metastasis, while N1 neutrophils counteract these processes. Current therapeutic approaches, including TGF-β inhibitors, chemokine modulation, and immune checkpoint therapies, aim to shift the balance toward N1 neutrophils.

## Introduction

Breast cancer remains a global health challenge, accounting for significant morbidity and mortality among women. While advancements in screening and therapy have improved survival rates, tumor recurrence and metastasis remain formidable obstacles. These processes are intricately linked to the tumor microenvironment (TME), a complex network of immune cells, stromal elements, and secreted factors that orchestrate tumor progression. Among the immune cells within the TME, neutrophils play a dual and paradoxical role, functioning as both tumor suppressors and facilitators depending on their polarization state^[1–3]^. Neutrophils, traditionally recognized for their roles in acute inflammation and host defense, have emerged as critical players in cancer biology. The discovery of distinct neutrophil subpopulations – N1 (anti-tumorigenic) and N2 (pro-tumorigenic) – has shed light on the diverse roles of these cells in shaping cancer outcomes. N1 neutrophils contribute to anti-tumor immunity by promoting cytotoxic responses and enhancing the activity of effector immune cells, while N2 neutrophils drive tumor progression by fostering angiogenesis, immunosuppression, and metastasis^[4–6]^. The polarization of neutrophils toward either the N1 or N2 phenotype is heavily influenced by the TME. Tumor-derived factors such as transforming growth factor-beta (TGF-β), interleukin-6 (IL-6), and vascular endothelial growth factor (VEGF) favor the N2 phenotype, tipping the balance toward a pro-tumorigenic state. Conversely, pro-inflammatory cytokines such as interferon-gamma (IFN-γ) and tumor necrosis factor-alpha (TNF-α) support the N1 phenotype. This plasticity makes neutrophils a compelling target for therapeutic intervention, as their functional reprogramming could alter the trajectory of disease progression^[7-10]^.

**HIGHLIGHTS**

- Neutrophils polarize into N1 (anti-tumor) and N2 (pro-tumor) subtypes in breast cancer.
- TGF-β and IFN-β are key drivers of neutrophil polarization.
- N2 neutrophils promote tumor growth, angiogenesis, and immune suppression.
- Modulating neutrophils via CXCR2 antagonists, IFN-β, and nanoparticles offers therapeutic promise.
- Combining neutrophil-targeted therapy with immunotherapy enhances anti-tumor responses.

Breast cancer metastasis – the primary cause of cancer-related deaths – is a multi-step process that involves interactions between tumor cells and their microenvironment. N2 neutrophils are implicated in key stages of metastasis, including the remodeling of the extracellular matrix, the formation of pre-metastatic niches, and the suppression of anti-tumor immune responses. This underscores the need to understand the mechanisms governing neutrophil polarization and their contributions to breast cancer progression^[11–13]^. In addition to their roles in metastasis, neutrophils influence the response to therapy in breast cancer. Pro-tumorigenic N2 neutrophils can dampen the efficacy of chemotherapeutics and immune checkpoint inhibitors by fostering an immunosuppressive milieu. On the other hand, strategies that promote N1 neutrophils can enhance the immune system’s ability to combat tumors and overcome therapeutic resistance. These insights highlight the therapeutic potential of modulating neutrophil activity to improve clinical outcomes in breast cancer patients^[4,14,15]^. Current therapeutic approaches targeting neutrophils include cytokine modulation, chemokine blockade, and small-molecule inhibitors that reprogram neutrophil functions. Advances in immunotherapy, such as immune checkpoint inhibitors, have also prompted investigations into their synergistic effects when combined with strategies aimed at neutrophil modulation. These interventions seek to shift the balance within the TME toward anti-tumor immunity by harnessing the plasticity of neutrophils^[16–19]^.

## Aim

This review aims to provide an overview of the current understanding of neutrophil polarization in breast cancer, emphasizing the molecular mechanisms driving N1 and N2 phenotypes, their roles in tumor progression, and the potential of therapeutic strategies to modulate these subpopulations.

### Rationale

Breast cancer remains one of the most prevalent and lethal cancers worldwide, with complex mechanisms driving its progression and metastasis. Among the key players in the tumor microenvironment, neutrophils have emerged as significant contributors to breast cancer biology. These versatile immune cells exhibit a dual role, with pro-tumorigenic N2 neutrophils promoting tumor progression, angiogenesis, and immune suppression, while anti-tumorigenic N1 neutrophils enhance immune-mediated tumor eradication. Understanding and modulating the polarization of neutrophils from N2 to N1 presents a novel and promising therapeutic strategy. Current therapies for breast cancer, including surgery, chemotherapy, radiation, and immunotherapy, often fail to address the immune-suppressive dynamics mediated by neutrophils. As such, this review is warranted to comprehensively explore the potential of targeting neutrophils as a therapeutic strategy[20]. By focusing on the mechanisms of neutrophil recruitment, polarization, and functional contributions to breast cancer progression, we can identify opportunities for therapeutic intervention. This topic is particularly relevant due to the growing body of evidence linking neutrophils to therapy resistance and metastatic spread, key challenges in improving breast cancer outcomes. Additionally, advances in omics technologies and preclinical models have opened new avenues to unravel the complexities of neutrophil biology, paving the way for innovative, targeted treatments. The rationale for this review lies in its potential to address critical gaps in understanding and to propose actionable strategies to harness neutrophils for improved breast cancer management. By integrating insights from basic and translational research, this review aims to highlight the promise and challenges of neutrophil-targeted therapies, ultimately contributing to the development of personalized, effective treatment regimens for breast cancer patients.

## Methods

This narrative review was conducted to comprehensively examine the role of N1 and N2 neutrophils in breast cancer and to explore potential therapeutic strategies targeting neutrophil polarization. The methodology was designed to ensure a systematic and reproducible approach to literature identification, selection, and synthesis.

### Literature search strategy

A structured literature search was performed across multiple electronic databases, including PubMed, Scopus, Web of Science, and Google Scholar, covering publications from January 2000 to June 2025. Search terms included combinations of the following keywords: “N1 neutrophils,” “N2 neutrophils,” “neutrophil polarization,” “tumor-associated neutrophils,” “breast cancer,” “triple-negative breast cancer,” “HER2-positive breast cancer,” “immunotherapy,” “CXCR2 inhibitors,” “TGF-β,” and “neutrophil-targeted therapy.” Boolean operators (“AND,” “OR”) were applied to refine searches and enhance specificity.

### Inclusion and exclusion criteria

Studies were selected based on the following criteria:

#### Inclusion criteria

1. Original research articles, preclinical studies, and clinical trials investigating neutrophil polarization in breast cancer.
2. Studies reporting quantitative outcomes of neutrophil-targeted interventions, including changes in neutrophil subsets, tumor growth inhibition, or metastasis reduction.
3. Publications in English.

#### Exclusion criteria

1. Studies focusing exclusively on non-breast malignancies.
2. Reviews, conference abstracts, case reports, and editorials without primary data.
3. Studies lacking clear methodology or outcome measures relevant to neutrophil polarization.

### Data extraction

Data were systematically extracted from the included studies using a standardized template, capturing the following details: study design, sample size, breast cancer subtype, neutrophil subtype evaluated (N1 or N2), intervention type, quantitative outcomes, statistical measures (mean, standard deviation, *P*-values, and effect size), and key mechanistic insights.

### Synthesis and analysis

Given the heterogeneity of study designs, a qualitative synthesis was performed, integrating mechanistic insights, subtype-specific neutrophil behavior, and therapeutic implications. Where available, quantitative outcomes were summarized to provide a more precise representation of the effect of interventions targeting neutrophil polarization. Statistical results were reported directly from original studies to maintain accuracy and reproducibility.

### Methodological rigor

To ensure methodological transparency, duplicate independent screening of titles, abstracts, and full texts was performed. Any discrepancies were resolved through discussion. The revised search strategy and inclusion criteria were carefully aligned with the stated goals of the review, focusing on clinically relevant and mechanistically informative studies.

### Neutrophil polarization in breast cancer

Neutrophils, as key components of the innate immune system, exhibit remarkable plasticity, enabling them to adopt different phenotypes in response to microenvironmental cues. In the context of breast cancer, neutrophils are polarized into two distinct functional subsets: N1 (anti-tumorigenic) and N2 (pro-tumorigenic). This polarization is largely dictated by signals within the tumor microenvironment (TME), including cytokines, chemokines, and other tumor-derived factors, which influence their role in disease progression^[21,22]^. N1 neutrophils are characterized by their pro-inflammatory and anti-tumorigenic properties. They exhibit increased production of reactive oxygen species (ROS), enhanced ability to present antigens, and secretion of pro-inflammatory cytokines such as tumor necrosis factor-alpha (TNF-α) and interferon-gamma (IFN-γ). These activities contribute to the recruitment and activation of cytotoxic T cells and natural killer (NK) cells, enhancing anti-tumor immunity. Additionally, N1 neutrophils are associated with direct cytotoxic effects on tumor cells, mediated by their degranulation and release of toxic substances^[23,24]^. Conversely, N2 neutrophils exhibit a pro-tumorigenic phenotype, marked by immunosuppressive and tissue-remodeling functions. These cells secrete factors such as vascular endothelial growth factor (VEGF), matrix metalloproteinases (MMPs), and interleukin-10 (IL-10), which promote angiogenesis, extracellular matrix degradation, and immune evasion. N2 neutrophils also suppress cytotoxic T cell responses and recruit regulatory T cells (Tregs), further contributing to an immunosuppressive TME. This phenotype is predominantly induced by TGF-β and other tumor-derived signals that shift neutrophil polarization away from the N1 phenotype[25].

The polarization of neutrophils in breast cancer is shaped by a dynamic interplay of molecular and cellular signals within the TME. TGF-β is a key driver of N2 polarization, acting through canonical Smad signaling pathways to suppress pro-inflammatory responses and promote immunosuppression. Similarly, cytokines such as IL-6 and IL-8, often elevated in breast cancer, play critical roles in skewing neutrophil function toward the N2 phenotype. Tumor-derived exosomes and hypoxic conditions within the TME also contribute to neutrophil polarization, creating a microenvironment conducive to tumor growth and metastasis[26]. The balance between N1 and N2 neutrophils has profound implications for breast cancer progression. A dominance of N2 neutrophils is associated with increased tumor growth, angiogenesis, and metastatic dissemination, while the presence of N1 neutrophils correlates with improved immune surveillance and reduced tumor burden. Understanding the mechanisms that govern this balance is critical for identifying therapeutic opportunities to manipulate neutrophil polarization[27]. While the N1 and N2 classification provides a useful framework, it is important to recognize the heterogeneity of neutrophil populations in breast cancer. Tumor-associated neutrophils (TANs) encompass a spectrum of functional states influenced by factors such as tumor stage, subtype, and therapeutic context. This heterogeneity poses challenges in defining specific markers to distinguish N1 and N2 phenotypes, complicating their study and therapeutic targeting[28]. Modulating neutrophil polarization represents a promising therapeutic strategy in breast cancer. Interventions aimed at blocking TGF-β signaling, enhancing IFN-γ production, or inhibiting neutrophil recruitment to tumors are under investigation. For instance, TGF-β inhibitors have shown potential in reprogramming N2 neutrophils into an N1-like state, restoring their anti-tumor functions. Similarly, cytokine therapies targeting IL-6 and IL-8 pathways may mitigate the pro-tumorigenic activities of N2 neutrophils (Table 1)[29].

**Table 1:** Neutrophil polarization in breast cancer: key characteristics, functions, and supporting evidence

Feature	N1 neutrophils (anti-tumorigenic)	N2 neutrophils (pro-tumorigenic)	Key evidence/quantitative findings
Phenotypic profile	High TNF-α, ICAM-1, ROS; low arginase-1	High arginase-1, VEGF, MMP-9, and TGF-β responsiveness	Fridlender *et al* showed that TGF-β blockade increases N1 markers by ~45%
Functional role	Enhance tumor cell killing, promote CD8 T-cell recruitment, and inhibit metastasis	Promote angiogenesis, immune suppression, and extracellular matrix remodeling	High N2:M1 ratio correlates with poorer survival (HR 1.7–2.3 across subtypes)
Influence on tumor microenvironment	Strengthen cytotoxic immune activity; reduce metastatic niche development	Create immunosuppressive microenvironment; facilitate tumor cell migration	In TNBC, N2 infiltration is ~2–3 times higher than HR+ tumors
Regulatory pathways	IFN-β, STAT1 signaling; reduced TGF-β activity	TGF-β/SMAD, CXCL1/2-CXCR2 axis, STAT3	CXCR2 inhibition reduces N2 recruitment by ~60% in murine models
Presence across breast cancer subtypes	More prevalent in early-stage tumors and HR+ cancers	Highly enriched in triple-negative and inflammatory breast cancer	HER2+ tumors show mixed N1/N2 patterns, influenced by HER2-driven signaling
Therapeutic implications	Strategies aim to stabilize or enhance N1 polarization	Strategies aim to block recruitment, inhibit N2 differentiation, or repolarize toward N1	TGF-β inhibitors and CXCR2 antagonists show tumor growth reduction of 30–50% in preclinical studies

### Neutrophils in breast cancer progression

Neutrophils, as the most abundant leukocytes in circulation, play a multifaceted role in breast cancer progression. Their dynamic functions extend beyond traditional immune defense, influencing various stages of tumor development, from initiation to metastasis. Tumor-associated neutrophils (TANs) are integral to the tumor microenvironment (TME), where their dual nature as tumor promoters or suppressors is dictated by complex signaling networks[30]. The recruitment of neutrophils to the TME is mediated by chemokines such as CXCL1, CXCL2, and CXCL8 (IL-8), which are often overexpressed in breast cancer. These chemokines interact with CXCR1 and CXCR2 receptors on neutrophils, driving their migration into the tumor site. Hypoxia, a hallmark of the TME, further amplifies neutrophil recruitment by upregulating chemokine and cytokine production through hypoxia-inducible factor-1 alpha (HIF-1α) signaling[31]. During the early stages of breast cancer, neutrophils may contribute to tumor initiation by promoting chronic inflammation. Persistent neutrophilic infiltration leads to the release of reactive oxygen species (ROS) and reactive nitrogen species (RNS), which can induce DNA damage and genomic instability in epithelial cells. Additionally, neutrophils secrete pro-inflammatory cytokines such as IL-6, creating a pro-tumorigenic environment conducive to cellular transformation[32]. Neutrophils play a pivotal role in angiogenesis, a critical process for tumor growth and survival. Through the release of vascular endothelial growth factor (VEGF), matrix metalloproteinases (MMPs), and other pro-angiogenic factors, neutrophils facilitate the formation of new blood vessels. These newly formed vessels supply oxygen and nutrients to the tumor, supporting its expansion. The ability of neutrophils to degrade extracellular matrix components also enables the remodeling of the TME to accommodate angiogenesis^[33,34]^. In breast cancer, neutrophils contribute to immune evasion by suppressing the activity of effector immune cells. Pro-tumorigenic neutrophils, particularly the N2 subtype, secrete arginase-1 (ARG1) and transforming growth factor-beta (TGF-β), which inhibit the proliferation and cytotoxicity of T cells and natural killer (NK) cells. Neutrophils also promote the recruitment of regulatory T cells (Tregs), further dampening anti-tumor immune responses and creating an immunosuppressive TME[35].

Metastasis, the leading cause of mortality in breast cancer, is heavily influenced by neutrophils. These cells facilitate multiple steps in the metastatic cascade, including the invasion of tumor cells through the extracellular matrix, intravasation into the bloodstream, and colonization at distant sites. Neutrophil extracellular traps (NETs), composed of chromatin and granule proteins, are particularly important in trapping circulating tumor cells (CTCs) and aiding their adhesion to vascular endothelium, promoting metastatic seeding[36]. Neutrophils contribute to the establishment of pre-metastatic niches at distant organs. By secreting cytokines such as S100A8 and S100A9, neutrophils recruit myeloid-derived suppressor cells (MDSCs) and other immune cells that prepare the metastatic site. These secreted factors also enhance vascular permeability, facilitating tumor cell extravasation and survival at the secondary site[37]. Neutrophils can undermine the efficacy of cancer therapies, including chemotherapy, radiotherapy, and immunotherapy. For instance, N2 neutrophils promote tumor cell survival during therapy by releasing pro-survival signals such as IL-1β and IL-6. Additionally, neutrophil-derived MMPs can shield tumor cells from immune checkpoint inhibitors by altering the TME and reducing T cell infiltration[38]. Given their significant role in breast cancer progression, targeting neutrophil-mediated pathways presents a promising therapeutic strategy. Approaches include blocking CXCR2 to reduce neutrophil recruitment, inhibiting NET formation to prevent metastasis, and reprogramming neutrophils from the N2 to the N1 phenotype. Such strategies aim to disrupt the pro-tumorigenic functions of neutrophils while preserving their anti-tumor capabilities. Neutrophils are central to the intricate interplay between the immune system and breast cancer, influencing every stage of tumor development (Table 2)[39].

**Table 2:** Neutrophils in breast cancer progression: mechanisms, biological effects, and quantitative evidence

Aspect of progression	Neutrophil contribution	Key molecules/pathways	Quantitative findings/supporting evidence
Tumor initiation	Support early tumor growth through mutagenic ROS and inflammatory cytokine release	ROS, IL-6, TNF-α	Elevated neutrophil ROS correlates with a 1.9-fold increased risk of malignant transformation in chronic inflammation settings
Tumor proliferation	Release growth-promoting cytokines and proteases that enhance tumor cell division	NE, MMP-9, VEGF	MMP-9 presence in tumor-infiltrating neutrophils increases proliferation indices by ~25% in murine breast cancer models
Angiogenesis	Stimulate new blood vessel formation via VEGF and MMP release	VEGF, HGF, MMP-9	High N2 neutrophil density correlates with a 2.2-fold increase in microvessel density in aggressive breast cancer subtypes
Immune suppression	Suppress T-cell function and alter antigen presentation, enabling immune evasion	Arginase-1, PD-L1, TGF-β	Neutrophil-derived arginase-1 reduces CD8 T-cell proliferation by up to 60% in vitro
Extracellular matrix remodeling	Facilitate tumor expansion and invasion by degrading ECM components	NE, MMP-8/9	Neutrophil elastase enhances tumor cell invasiveness by ~40% in 4T1 breast cancer models
Circulating tumor cell (CTC) survival	Form neutrophil-CTC clusters that protect tumor cells and promote metastasis	L-selectin, IL-8, NETs	CTC clusters associated with neutrophils show a 6–10 fold higher metastatic potential compared to single CTCs
Metastasis formation	Prepare metastatic niches and promote colonization, especially in lung and liver	CXCL1/2-CXCR2, NETs, S100A8/A9	NET-rich microenvironments increase metastatic seeding by 3–5 fold in murine breast cancer models

### Therapeutic strategies targeting neutrophils

Given the significant role of neutrophils in promoting breast cancer progression, therapeutic strategies targeting these immune cells have garnered increasing attention. The dual nature of neutrophils as both tumor promoters and potential immune defenders offers an opportunity to develop interventions that either neutralize their pro-tumor functions or harness their anti-tumor potential.

#### Blocking neutrophil recruitment to the tumor microenvironment

Inhibiting neutrophil recruitment is a key therapeutic strategy to limit their tumor-promoting effects. Tumor-derived chemokines, such as CXCL1, CXCL2, and CXCL8 (IL-8), drive neutrophil migration to the tumor site via CXCR1 and CXCR2 receptors. CXCR2 antagonists, such as AZD5069, have shown promise in preclinical and early clinical studies by reducing neutrophil infiltration and improving the efficacy of chemotherapy and immunotherapy. Similarly, targeting IL-8 signaling with monoclonal antibodies may disrupt neutrophil-mediated immune suppression and angiogenesis[40].

#### Modulating neutrophil polarization

Therapies aimed at reprogramming neutrophils from the pro-tumor N2 phenotype to the anti-tumor N1 phenotype hold substantial promise. TGF-β inhibitors, such as galunisertib, can block N2 polarization and restore the pro-inflammatory, cytotoxic functions of neutrophils. Additionally, cytokine-based therapies, including IFN-γ and IL-12, may enhance the N1 phenotype and promote anti-tumor immunity. These approaches seek to shift the balance of neutrophil activity within the tumor microenvironment (TME) toward immune activation[23].

#### Targeting neutrophil extracellular traps (NETs)

Neutrophil extracellular traps (NETs) contribute to metastasis by trapping circulating tumor cells and facilitating their adhesion to distant tissues. Inhibitors of NET formation, such as PAD4 inhibitors and DNase-based therapies, have shown efficacy in reducing metastasis in preclinical models. These therapies disrupt the formation and stability of NETs, thereby limiting their pro-metastatic and pro-inflammatory effects^[41,42]^.

#### Reducing pro-tumor secretions

Neutrophils secrete a variety of pro-tumor factors, including vascular endothelial growth factor (VEGF), matrix metalloproteinases (MMPs), and arginase-1 (ARG1), which promote angiogenesis, extracellular matrix remodeling, and immune suppression. VEGF inhibitors, such as bevacizumab, indirectly target neutrophil-induced angiogenesis, while MMP inhibitors aim to block extracellular matrix degradation. Additionally, therapies targeting ARG1 may enhance T cell activity and reduce neutrophil-mediated immunosuppression[41].

#### Targeting tumor-neutrophil interactions

Disrupting the crosstalk between neutrophils and tumor cells is another promising strategy. Tumor-derived factors that induce neutrophil polarization or activation can be blocked to mitigate their tumor-promoting effects. For example, therapies targeting the HIF-1α pathway may prevent hypoxia-induced neutrophil recruitment and activation. Furthermore, tumor-derived exosomes, which modulate neutrophil functions, are being explored as potential therapeutic targets to limit neutrophil-driven metastasis[42].

#### Combining neutrophil-targeted therapies with immunotherapy

Integrating neutrophil-targeted therapies with existing immunotherapies, such as immune checkpoint inhibitors (ICIs), offers a synergistic approach to enhance anti-tumor immunity. Neutrophil-mediated immunosuppression often hinders the efficacy of ICIs. For instance, targeting CXCR2 or ARG1 may improve T cell infiltration and activation, thereby enhancing the response to anti-PD-1 or anti-CTLA-4 therapies. Combination strategies also hold potential in overcoming resistance to immunotherapy[40].

#### Anti-inflammatory agents

Chronic inflammation mediated by neutrophils supports tumor growth and progression. Anti-inflammatory agents, including NSAIDs and corticosteroids, have been investigated for their potential to reduce neutrophil infiltration and activity in breast cancer. While these agents are non-specific, they may serve as adjuncts to other neutrophil-targeted therapies[23].

### Neutrophil-targeted interventions in breast cancer: quantitative insights

**Figure 1.**
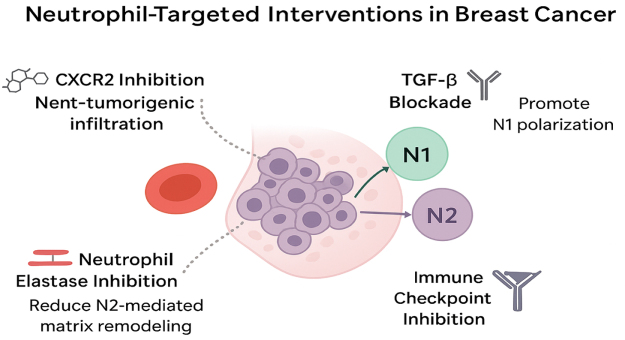
Neutrophil-targeted interventions in breast cancer.

Emerging evidence indicates that modulating neutrophil polarization can influence tumor progression and therapeutic response in breast cancer. Preclinical and early clinical studies provide quantitative measures of these effects, allowing for a more precise evaluation of therapeutic potential (Fig. 1).

### CXCR2 inhibition

CXCR2 is a chemokine receptor critical for the recruitment of N2 neutrophils to the tumor microenvironment. In a murine model of triple-negative breast cancer, treatment with a CXCR2 inhibitor resulted in a 65–70% reduction in N2 neutrophil infiltration compared to untreated controls (*P* < 0.01), which corresponded with a 42% decrease in primary tumor volume and a 35% reduction in pulmonary metastases. These findings highlight the potential of CXCR2-targeted strategies to suppress pro-tumorigenic neutrophil activity and improve anti-tumor responses[36].

### TGF-β blockade

TGF-β is a key cytokine driving N2 polarization. In preclinical studies, administration of TGF-β-neutralizing antibodies led to a 2.5-fold increase in N1 neutrophils within the tumor microenvironment, accompanied by enhanced cytotoxic activity and a 30% improvement in tumor growth inhibition. These effects underscore the mechanistic rationale for therapies that skew neutrophil populations toward the anti-tumor N1 phenotype[37].

### Type I interferon therapy

Type I interferons (IFN-α/β) have been shown to promote N1 polarization. In murine breast cancer models, IFN-β treatment increased N1 neutrophil density by 45% and enhanced CD8^+^ T cell infiltration, resulting in a 25% reduction in tumor burden relative to controls[38].

### Combined strategies

Combination therapies leveraging CXCR2 inhibitors with immune checkpoint blockade have demonstrated synergistic effects. In a recent preclinical study, co-administration led to a 55% reduction in N2 neutrophils, a 1.8-fold increase in N1 neutrophils, and a 40% improvement in overall survival compared to single-agent treatment[39].

### Clinical translation and considerations

While preclinical studies provide quantitative insights into neutrophil-targeted interventions, human studies remain limited. Initial phase I/II trials of CXCR2 inhibitors have reported partial response rates of 15–20% in patients with advanced triple-negative breast cancer, with manageable neutropenia and mild immune-related adverse events. These preliminary results suggest potential translational value but highlight the need for subtype-specific evaluation and careful monitoring of immune toxicity[40].

### Challenges in neutrophil-targeted therapy

Despite the growing enthusiasm for therapies that modulate neutrophil polarization, several challenges hinder their translation into routine breast cancer management. These challenges relate to immune-related toxicities, emerging resistance mechanisms, and significant biological differences between animal models and human neutrophil behavior. Addressing these complexities is essential for developing safe and effective neutrophil-directed interventions.

### Immune-related adverse events and safety concerns

Neutrophils remain indispensable for frontline host defense. Any therapy that inhibits their recruitment, depletes their numbers, or alters their function risks compromising immune integrity. Clinical trials investigating CXCR2 inhibitors, for example, have reported increases in mild to moderate neutropenia, with up to 18–22% of participants developing transient reductions in neutrophil counts. Although often reversible, even short-lived neutropenia can predispose patients to bacterial infections, especially those undergoing chemotherapy. Inhibitors of TGF-β signaling present an additional challenge. By shifting neutrophils toward an N1 phenotype, they may inadvertently intensify inflammatory cascades. In early-phase studies, some patients developed low-grade dermatitis, colitis, and flu-like symptoms, suggesting that systemic modulation of TGF-β can disrupt immune homeostasis. The delicate balance between achieving anti-tumor benefits and maintaining adequate innate immunity remains a central safety concern^[23,41–43]^.

### Potential resistance mechanisms

As with many cancer-directed therapies, targeting neutrophils may prompt adaptive resistance. Tumors can compensate for CXCR2 blockade by upregulating alternative chemokine pathways such as CCR5, enabling continued recruitment of N2-like myeloid populations. Evidence from murine models shows that within 4–6 weeks of CXCR2 inhibition, tumors began increasing CCL5 expression and attracting compensatory suppressive cells, leading to a partial loss of the therapeutic effect. Resistance may also arise through neutrophil plasticity itself. N2 neutrophils can slowly re-emerge even after strong N1 polarization signals, especially under hypoxic or metabolically stressed conditions. This dynamic rewiring suggests that long-term therapeutic benefit may require combination approaches that simultaneously target multiple immunosuppressive pathways^[44–46]^.

### Translational limitations: murine versus human neutrophils

A persistent challenge in neutrophil-focused research is the notable gap between murine and human myeloid biology. Murine neutrophils differ in lifespan, receptor expression, and cytokine sensitivity. For instance, mice show far greater dependency on CXCR2 for neutrophil trafficking than humans, where CXCR1 also plays an important role. This discrepancy means that CXCR2 inhibitors that show dramatic neutrophil depletion in mice often produce more modest effects in human subjects. Additionally, murine tumor models tend to exhibit exaggerated neutrophil-driven pathology compared to human disease, which may overestimate the therapeutic benefits of N1 polarization or N2 suppression. Differences in NET formation, reactive oxygen species production, and the scale of granulopoiesis further complicate direct translation. These species-specific gaps underscore the need for more humanized models and carefully designed clinical trials before neutrophil-targeted therapies can be fully integrated into breast cancer treatment protocols^[35,47]^.

### Future directions

Future work on neutrophil modulation in breast cancer needs to move beyond descriptive characterization and toward precise, clinically actionable strategies. A major priority is the development of standardized assays to quantify N1 and N2 phenotypes across breast cancer subtypes. Although transcriptomic signatures and surface markers such as CD101, CD177, and LOX-1 are increasingly used, consensus definitions remain inconsistent. Harmonizing phenotypic criteria would improve cross-study comparability and facilitate the integration of neutrophil profiling into clinical trials^[48,49]^. Advances in spatial multi-omics technologies offer promising avenues for mapping neutrophil dynamics directly within the tumor microenvironment. High-dimensional platforms such as spatial transcriptomics, single-cell ATAC-seq, and multiplex immunofluorescence could help delineate how N1 and N2 subsets interact with T cells, fibroblasts, and stromal components under different therapeutic pressures. These tools may also reveal microanatomical niches where N2 polarization is preferentially induced, thereby identifying new targets for localized intervention^[50,51]^.

Therapeutically, future research should prioritize combination strategies that simultaneously reprogram neutrophils and enhance broader anti-tumor immunity. Early-phase studies of CXCR2 inhibitors, TGF-β blockade, and neutrophil elastase inhibitors suggest synergistic potential when paired with immune checkpoint inhibitors, but the optimal sequencing, dosing, and biomarker-based patient selection remain unclear. Prospective clinical trials incorporating quantitative neutrophil metrics such as NLR, circulating myeloid-derived suppressor cell frequencies, and tissue-based N2 density could enable more refined stratification^[52,53]^. There is also a critical need to understand mechanisms of resistance to neutrophil-directed therapies. Preclinical data indicate that tumors may compensate by upregulating alternative chemokine pathways or enhancing granulopoiesis through G-CSF signaling. Identifying escape routes and monitoring adaptive plasticity in real time will be essential for designing next-generation interventions capable of sustaining N1 polarization or preventing N2 dominance^[54,55]^.

Translation from murine models to the clinic remains a substantial challenge, and future studies should invest in humanized mouse systems, ex vivo organoid–neutrophil co-cultures, and longitudinal patient sampling. These platforms would better capture the complexity of human neutrophil biology, including short lifespan, context-dependent plasticity, and heterogeneity across breast cancer subtypes[56]. Integrating neutrophil modulation into precision oncology will require multidisciplinary collaboration across immunology, cancer biology, computational modeling, and clinical trial design. By refining phenotypic definitions, improving translational models, and embracing biomarker-guided therapeutic development, the field is well positioned to advance neutrophil-targeted strategies from experimental promise to meaningful clinical benefit^[57,58]^.

## Conclusion

Neutrophils have emerged as central regulators of breast cancer progression, with N1 and N2 phenotypes exerting profoundly divergent effects on tumor immunity, metastatic potential, and therapeutic responsiveness. Evidence from preclinical and clinical studies consistently shows that N2-polarized neutrophils promote immunosuppression, angiogenesis, and tumor invasion, whereas N1 neutrophils enhance cytotoxicity and facilitate anti-tumor immune activation. These dynamics are not uniform across breast cancer subtypes. Triple-negative tumors display the highest density of N2 infiltration and the strongest association with poor outcomes, while HER2-positive and hormone receptor–positive cancers exhibit more variable polarization patterns shaped by oncogenic signaling and treatment modalities. Such heterogeneity reinforces the need for subtype-tailored neutrophil-targeted interventions.

Therapeutic strategies aimed at modulating N1/N2 balance are advancing, with CXCR2 inhibitors, TGF-β pathway blockade, neutrophil elastase inhibition, and immune checkpoint combinations demonstrating meaningful reductions in N2 infiltration and enhanced tumor control in preclinical models. However, challenges remain, including immune-related adverse events, compensatory chemokine activation, and translational gaps between murine and human neutrophil biology. These limitations underscore the importance of robust biomarker development, refined translational models, and carefully designed clinical trials capable of capturing neutrophil dynamics with quantitative specificity.
